# Exploring adsorption dynamics of heavy metals onto varied commercial microplastic substrates: Isothermal models and kinetics analysis

**DOI:** 10.1016/j.heliyon.2024.e35364

**Published:** 2024-07-29

**Authors:** Anda-Gabriela Tenea, Cristina Dinu, Paul Alexandru Rus, Ioana Alexandra Ionescu, Stefania Gheorghe, Vasile Ion Iancu, Gabriela Geanina Vasile, Luoana Florentina Pascu, Florentina Laura Chiriac

**Affiliations:** National Research and Development Institute for Industrial Ecology ECOIND Bucharest, 51-73 Drumul Podul Dambovitei Street, 060652, Bucharest, Romania

**Keywords:** Heavy metals, Adsorption, Microplastics, Isotherm, Kinetics

## Abstract

The increasing presence of plastics in the environment has raised concerns about their potential impact, especially as carriers of heavy metals such as Cd, Ni, and Pb. However, the adsorption mechanism of heavy metals on microplastics remains poorly understood. In this study, we investigated the adsorption behavior of Cd, Ni, and Pb by polystyrene (PS) and polypropylene (PP) microplastics to better comprehend their interaction and potential environmental implications. Our results revealed that equilibrium adsorption of microplastics with different heavy metals was achieved within a 6-h contact time. The FTIR analysis findings, which suggest that physical interactions play a significant role in the adsorption of heavy metals onto microplastics, are further supported by the observed changes in surface morphology after adsorption. We explored the influence of solution pH, contact duration, and initial concentration on the adsorption capacity and found significant effects on the adsorption behavior. To model the adsorption process, we applied Langmuir and Freundlich adsorption isotherm models and observed that the Langmuir model better fit the experimental data. Furthermore, we compared the pseudo-first and pseudo-second-order kinetic models and found that the pseudo-second-order model provided a more accurate description of the adsorption kinetics. Notably, the adsorption percentages varied depending on the type of microplastic and experimental conditions. Overall, this study enhances our understanding of the adsorption mechanism of heavy metals on microplastics and provides valuable insights into their behavior in aquatic environments. These findings have implications for the development of effective strategies for mitigating pollution caused by microplastics and heavy metals in aquatic ecosystems.

## 1 Introduction

The presence of microplastics (MPs) in the environment is a globally recognized issue. MPs are tiny pieces of plastic, measuring less than 5 mm in diameter. They can be categorized into two types: primary and secondary. Primary MPs are intentionally manufactured and can be found in products like facial cleansers (microbeads) and resin particles. Secondary microplastic particles are produced as a result of the aging, weathering, and biodegradation processes that cause larger plastic waste items to break down. The studies have found significant amounts of microplastics in the sediments of the tropical Atlantic Ocean and China's largest lakes [1,2] Surprisingly, even areas with low population density and plastic consumption, such as the Qinghai-Tibet Plateau, have been found to have high levels of microplastics [3]. The widespread distribution of MPs in different ecosystems, including surface water and water bodies, has been well-documented [[4], [5], [6], [7]]. The pollution sources are due to human activities such as improper waste disposal and plastic debris fragmentation [8]. This highlights the potential for MPs to accumulate in these environments, presenting risks to living organisms [[9], [10], [11]]. The ingestion of MPs by aquatic organisms is of particular concern, as they may mistake these tiny plastic particles for food. Exposure to microplastics has been linked to negative impacts on feeding, reproduction, antioxidant defense, and innate immunity in aquatic organisms [12,13]. The potential transfer of MPs up the food chain raises ecological concerns and the potential for human exposure through the consumption of contaminated seafood and marine salt.

The issue of microplastic pollution and its associated risks to organisms requires a comprehensive understanding of the sources, distribution, and impacts of these particles on ecosystems and public health [14]. Further research is needed to develop effective mitigation strategies and to assess the long-term consequences of microplastics in aquatic ecosystems. Collaboration among researchers, policymakers, industries, and the public is essential to effectively tackle the challenges associated with MP pollution and preserve the integrity of our environment. Efforts to address the issue of MPs in the environment require a comprehensive approach that includes reducing plastic production and usage, improving waste management practices, and developing techniques to remove and mitigate the presence of MPs [15,16].

Another pollution problem comes from the presence of heavy metals in the environment, originate from both natural sources and human activities [8,17]. These non-degradable contaminants can continuously enter water bodies, leading to their enrichment and cycling within aquatic ecosystems. The combination of MPs and heavy metals presents a significant global threat, and raises concerns about the potential transfer of these contaminants to aquatic organisms in both marine and freshwater environments [18]. The physical characteristics of microplastics, including pore size, surface area, and type, have an impact on the adsorption of heavy metals onto them [19,20]. The MPs, due to their large surface area, can attract and accumulate toxic pollutants, acting as carriers for these contaminants and reaching high concentrations. The studies have identified traces of heavy metals on MPs in various locations worldwide. For example, heavy metals have been detected on MPs in the North Atlantic subtropical gyre, southeastern Brazil's São Paulo State, beaches in southwest England, and western Europe [[21], [22], [23], [24], [25]]. These studies indicated contamination of MPs with heavy metals and their potential for bioaccumulation and biomagnification [26]. For this reason, the coexistence of MPs and heavy metals underscores the need for comprehensive strategies to address both issues [21,22], such as implementing effective waste management strategies, reducing plastic use, and mitigating the release of heavy metals into aquatic environments [8,18]. It is crucial to consider the complex interactions between MPs and heavy metals to mitigate their potential risks and safeguard the health of ecosystems and human populations [27,28].

Previously, the interaction between metals and plastics was undetermined, due to a prevalent belief that polymers were not effective in their interaction with metals. However, recent studies have revealed the crucial role of microplastics in transporting metals in aquatic environments and their strong affinity for adsorbing heavy metals in freshwater systems [29]. Further research is needed to understand the extent of this interaction, the mechanisms of metal transfer, and the overall impacts on ecosystems, regarding their behaviour, fate, and transport in the aquatic environment. By examining these interactions, valuable insights can be gained into the risks associated with heavy metal contamination, bioaccumulation, and toxicity in aquatic ecosystems.

Romania has diverse aquatic ecosystems susceptible to microplastic and heavy metal contamination, with the Danube River being a significant international watercourse [30]. Understanding the fate and transport of microplastics and heavy metals in this region is crucial for ensuring its health and sustainability. Additionally, Romania's industrial and agricultural activities contribute to heavy metal pollution, making it essential to study the interactions between microplastics and heavy metals in the country. The research findings from this study can provide insights into the specific mechanisms and risks associated with heavy metal adsorption onto microplastics in Romanian aquatic ecosystems to develop effective pollution control strategies, management regulations, and environmental policies aligned with international protocols.

This study investigates the adsorption mechanisms of heavy metals (Cd, Ni, and Pb) onto two types of microplastics (polypropylene - PE and polystyrene - PS) in controlled laboratory conditions to address the critical research gap concerning microplastics and heavy metal pollution in aquatic environments. The heavy metals Cd, Ni, and Pb were chosen for this study due to their prevalent presence in aquatic environments and their significance as common pollutants with known adverse effects on ecosystems and organisms. The targeted metal ions are widely recognized for their toxicological properties, persistence in the environment, and their potential to bioaccumulate in aquatic organisms, posing serious risks to aquatic ecosystems. Previous studies indicated pollution of surface water with Cd, Ni, and Pb in different ecosystems from Romania [[31], [32], [33]]. The findings aim to guide the development of effective pollution control measures and management strategies to protect biodiversity and sustain water resources, ultimately contributing to the preservation of the health of aquatic environments and informing policy decisions for environmental sustainability.

## 2 Materials and methods

### 2.1 Materials and reagents

Standard solutions of Cd, Ni, and Pb at 1000 mg/L were purchased from CPA Chem (Bogomilovo, Bulgaria). Microplastic particles of PS and PP were supplied by Sigma-Aldrich (Darmstadt, Germany) and Goodfellow Cambridge Limited (Huntingdon, England), respectively. Hydrochloric acid (HCl) and sodium hydroxide (NaOH), purchased from Sigma-Aldrich (Darmstadt, Germany), were used for pH adjustment. The nitric acid (HNO_3_) used to rinse the glass vials was also procured from Sigma Aldrich (Darmstadt, Germany). The ultrapure water used in the experiment was obtained in-house using a Milli-Q station (Merk Millipore, Darmstadt, Germany).

### 2.2 Characterization methods for microplastics (MPs)

The surface functional groups of the MPs were determined using FTIR spectra analysis. FTIR Spectrum BX II PerkinElmer spectrophotometer (Waltham, MA, USA) was utilized for this purpose. The measurement was carried out within the range of 4000 and 800 cm^−1^. The microscopic morphology and surface structure of MP particles were investigated using a Scanning Electron Microscope (SEM) Quanta 250 FEG equipment, obtained from Thermo Fisher Scientific (Waltham, MA, USA).

### 2.3 Experimental design: Adsorption of heavy metals onto microplastics (MPs)

This study aimed to explore the impact of pH (ranging from 3 to 11), contact time (ranging from 0 to 24 h), and initial concentration (ranging from 0.2 to 10 mg/L) on the adsorption capacity of microplastics for heavy metals. These parameters play crucial roles in adsorption processes, as they can significantly affect both the solution and the characteristics of the adsorbent. The objective was to identify the optimal conditions for achieving maximum adsorption efficiency.

The batch adsorption experiment was conducted in a 200 mL solution of ultrapure water containing 10 mg/L of heavy metal. The experiment was carried out at room temperature, specifically 25 °C. To ensure consistent mixing, an automatic stirrer (IKA® KS 501 digital, Deutschland, Germany) was employed at a stirring speed of 100 rpm. After adding 0.5 g of MPs (microplastics), the system was stirred for various time intervals: 5, 10, 15, 30, 60, 120, 180, 240, 300, 360, and 1440 h (24 h). At each time interval, 10 mL samples were withdrawn using disposable syringes and filtered through 0.22 μm cellulose acetate filters. Subsequently, the collected samples were analyzed using an Avio 500 inductively coupled plasma optical emission spectrometer (ICP-EOS, manufactured by PerkinElmer, Waltham, Massachusetts, United States) to determine the remaining concentrations of heavy metals.

To investigate the effect of solution pH on the adsorption effectiveness of heavy metals onto MPs, the pH was controlled within the range of 3–11 using 0.01 M HCl and 0.01 M NaOH solutions as adjusting media. The experimental setup included a sample volume of 200 mL, a dosage of 0.5 g of MPs, a concentration of 10 mg/L for the heavy metals, and a contact time of 6 h.

The adsorption isotherm experiment proceeded by adding 0.5 g of MPs to 200 mL solutions containing heavy metals at different values (ranging from 0.2 to 10 mg/L) at a temperature of 25 °C. The microplastics that underwent the adsorption process were collected, cleaned with Milli-Q water, and subsequently dried at 35 °C for characterization purposes. To ensure accurate analysis, a blank control and three parallel control experiments were established.

By utilizing Equation (1), the adsorption capacity of MPs was calculated. Q_t_, denoting the total amount of heavy metals adsorbed onto MPs (in mg/g), was determined by subtracting the remaining concentration (C_t_) from the initial concentration (C_0_) of heavy metals in the solution (in mg/L), and then multiplying it by the solution volume (V) and the microplastic mass (m, in g).

(1) $$Q_{t}=\frac{\left(C_{0}-C_{t}\right)\cdot V}{m}$$

### 2.4 Adsorption isotherms

To understand how microplastics (MPs) adsorb heavy metals, various models have been applied to analyze metal adsorption isotherms. These models include the Langmuir, and Freundlich models. The Langmuir model assumes that the adsorbent surface has a single layer of coverage with uniform adsorption sites. It considers homogeneous adsorption, where all adsorption sites possess the same affinity for the adsorbate. This model also assumes fixed enthalpy and activation energy of adsorption within the adsorbent molecule. In a surface-plane adsorbate, migration does not occur. In contrast, the Freundlich model proposes that adsorption occurs on a surface that is heterogeneous in nature. It is suitable for both single-layer and multilayer adsorption processes. The affinity of the binding site varies, depending on the force between the adsorbent and the absorbent. These models, described by Equations (2), (3), (4), allow for a better understanding of the adsorption mechanisms of heavy metals onto MPs [[34], [35], [36], [37]].

(2) Q_e_ = (Q_m_ × K_L_ × C_e_) / (1+ K_L_ × C_e_)

(3) R_L_ = 1 / (1 + C_0_ × K_L_)

(4) Q_e_ = K_F_ × C_e_^1/n^

In the Langmuir model, Qe, which represents the adsorption capacity at equilibrium, is expressed in mg/g. The equilibrium concentration of the metal adsorbate is denoted by Ce and measured in mg/L. Qm, on the other hand, represents the maximum monolayer adsorption capacity in units of mg/g. Finally, the Langmuir equilibrium constant is denoted by K_L_ and expressed as L/mg, while R_L_ is the separation factor. The R_L_ value is used to determine the nature of adsorption, indicating whether it is favorable (0 < R_L_ < 1), unfavorable (R_L_ > 1), linear (R_L_ = 1), or irreversible (R_L_ = 0). The Freundlich model utilizes the Freundlich constants, KF and n, where 1/n is considered the heterogeneity factor. A value of n greater than 1 signifies favorable adsorption in the Freundlich model.

### 2.5 Adsorption kinetic model

The adsorption kinetic model enables the examination of the correlation between the duration required for a reaction to take place and the quantity of adsorbate that can be adsorbed. This analysis helps to understand the mechanism of heavy metal adsorption onto microplastics and identify the rate-limiting steps involved. The kinetic data in this study is analyzed using three models: the pseudo-first-order model (5), the pseudo-second-order model (6), and the intra-particle diffusion model (7). The mathematical equations for these models are provided below.

(5) Ln (Q_e_ − Q_t_) = LnQ_e_ − k_1_

(6) t/Q_t_ = 1 / (k_2_ × Q_e_^2^) + 1 / Q_e_

(7) Q_t_ = k_id_ × t^1/2^ + C_id_

The pseudo-first-order kinetic rate constant, denoted as k_1_ (min^−1^), is used in the pseudo-first-order kinetic model. The rate constant of the pseudo-second-order kinetic model is represented by k_2_ (g/(mg × min)). The intra-particle diffusion rate constant is k_id_ (mg/g min^1/2^), and the intra-particle diffusion constant is C_id_.

### 2.6 Quality control

Prior to the experiment, the glass vials needed for the study were immersed in 10 % HNO_3_ for 48 h to prevent any potential cross-contamination. Afterward, each vial underwent a thorough washing with Milli-Q water to ensure that no contaminants remained. To establish a starting point, a blank calibration control was performed three times for each concentration used in the experiment. This control involved measuring the solute concentration without any solid particles present. After achieving adsorption equilibrium, the concentration of the solute in the blank calibration control was determined. This measurement served as a reference point for the actual initial concentration, taking into account any loss caused by adsorption on the device's surface. Finally, the amount of solute adsorbed on the solid particles was calculated by determining the difference between the blank calibration control and the corresponding filtrate.

## 3 Results and discussion

### 3.1 Characterization of microplastics (MPs)

**Fig. 1:**
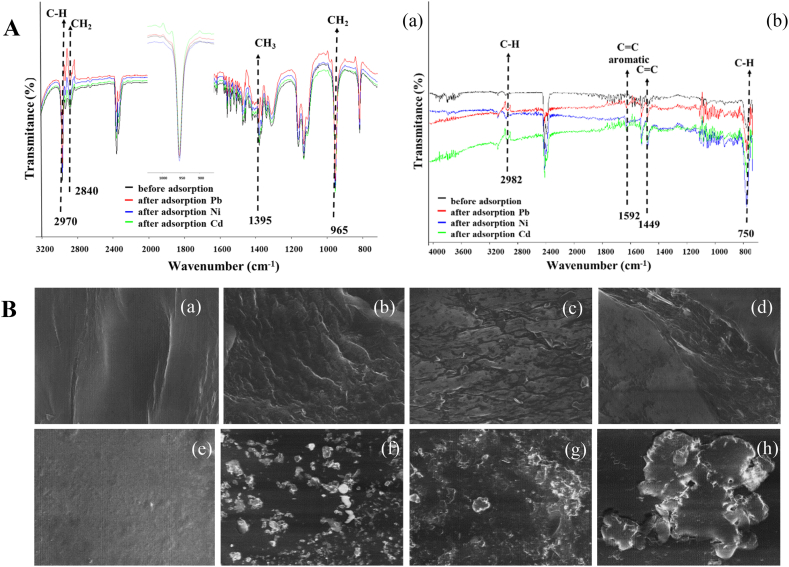
A. Fourier transform infrared (FTIR) spectra of (a) polypropylene (PP), and (b) polystyrene (PS) before adsorption and after adsorption. B. SEM images of (a) PP befor adsorption, (b) PP after Cd^2+^ adsorption, (c) PP after Ni^2+^ adsorption, (d) PP after Pb^2+^, (e) PS befor adsorption, (f) PS after Cd^2+^ adsorption, (g) PS after Ni^2+^ adsorption, (h) and PP after Pb^2+^ (recorded at scale 40 μm).

The infrared spectrum of PP in the range of 800–3200 cm^−1^ is depicted in Fig. 1A(a). The similar shape observed in the FTIR spectra of PP before and after adsorption suggests that the overall structure of the polymer remains unchanged after the adsorption of heavy metals on MPs. This indicates that the adsorption process does not cause significant structural alterations in the PP matrix. The presence of characteristic peaks at 2970 cm^−1^ (C–H stretching vibration), 2840 cm-1 (CH_2_ deformation), and 1385 cm^−1^ (CH_3_ symmetrical deformation) in both spectra confirms the presence of these functional groups in the PP sample. These peaks can be attributed to the polymer backbone and side chains, which are unaffected by the adsorption process. The absorption peak at 965 cm^−1^, assigned to –CH_3_ rocking vibration, further confirms the presence of methyl groups in the PP sample both before and after adsorption. This peak also remains unchanged, indicating that the adsorption of heavy metals does not significantly impact the methyl groups in the polymer. Overall, the FTIR analysis suggests that the primary chemical structure of PP remains intact during the adsorption process. The similarities in the FTIR spectra before and after adsorption indicate that any differences in functional groups are likely attributed to the interaction of heavy metal ions with the surface of the MPs rather than structural alterations in the PP polymer itself. This information is valuable in understanding the behavior of PP as a potential adsorbent for heavy metal removal from contaminated water or other environmental applications [7,38,39].

The FTIR analysis of PS before and after adsorption reveals that the functional groups of PS remain unchanged, indicating the presence of PS-specific functional groups. The characteristic peaks observed at 1592 cm^−1^ (aromatic ring), 1449 cm^−1^ (C=C bond in the aromatic ring), and 750 cm^−1^ (CH group in the aromatic ring) are consistent with the expected functional groups in PS. Notably, an absorption peak at 2982 cm^−1^ was also observed, which can be attributed to the aromatic C–H stretching vibration (Fig. 1A(b)). The absence of new characteristic peaks after adsorption suggests that heavy metals primarily adsorb onto the MPs through their intrinsic functional groups. This indicates that physical interactions, such as hydrophobic interactions, electrostatic forces, hydrogen bonding, and halogen bonding, are likely responsible for the adsorption process. However, the specific contributions of these different interaction mechanisms need to be further investigated to gain a comprehensive understanding of their role in heavy metal adsorption on MPs. The FTIR analysis and the absence of new peaks indicate that the adsorption process does not cause significant changes in the functional groups of PS, underscoring the stability of the polymer structure during heavy metal adsorption on MPs [7,40].

The FTIR results presented in Fig. 1A indicate that the adsorption of heavy metals onto microplastics is primarily driven by physical interactions. This aligns with previous studies that have highlighted the importance of forces such as electrostatic interactions, halogen bonding interactions, and π-π stacking in the adsorption process [41]. However, the chemical structure and intermolecular interactions of microplastics can influence the equilibrium adsorption capacity [42]. In a study [43] investigating the adsorption of antibiotics on microplastics, oxygen-containing functional groups, aromatic ring stacking interactions, and hydrogen bonding interactions were found to play significant roles in the adsorption process. Given the similarities in the functional groups and intermolecular interactions between antibiotics and heavy metals, it is reasonable to speculate that these factors may also contribute to the adsorption of metals on microplastics.

The presence of oxygen-containing functional groups on microplastics provides additional binding sites for metal ions through coordination or complexation reactions. π-π interactions, involving the stacking of aromatic rings of microplastics and metal ions, can enhance the adsorption capability by facilitating stronger surface interactions. Additionally, hydrogen bonding interactions between functional groups on microplastics and metal ions can also contribute to the adsorption process by stabilizing the adsorbed species. While physical interactions such as electrostatic forces, halogen bonding, and π-π stacking appear to be the dominating forces in the adsorption of heavy metals onto microplastics, the chemical structure and intermolecular interactions of microplastics, including oxygen-containing functional groups, π-π interactions, and hydrogen bonding, may also play significant roles [[41], [42], [43]] Further research is necessary to investigate the specific contributions of these interactions and fully comprehend their influence on the adsorption of heavy metals on microplastics.

The surface morphology of microplastics (MPs) plays a crucial role in determining their adsorption capabilities for heavy metals. In this study, SEM analysis was performed to assess the surface changes of MPs before and after adsorption of different heavy metals, as shown in Fig. 1B. The original MPs had smooth and regular surfaces, indicating a homogeneous surface without any significant features. However, noticeable changes in surface morphology were observed after the adsorption of heavy metals observed on the surface of the two studied microplastics. The formation of deposits on the microplastic surfaces indicates that the heavy metal ions have been adsorbed onto the MPs during the adsorption process, suggesting that the microplastic surface provides suitable sites for the adsorption of heavy metals. Moreover, the observed surface changes and deposits validate the findings from the FTIR analysis, indicating that physical interactions between the microplastic's functional groups and heavy metal ions play a significant role in the adsorption process.

### 3.2 Influence of pH

The most important factor in the adsorption of heavy metals is pH. During the experiments, the concentration and contact time were kept constant, and pH values ranging from 3 to 11 (3, 5, 7, 9, and 11) were used. At pH values higher than 5, the metal ions studied in the aqueous solutions precipitate as hydroxides. Therefore, at pH values higher than 5, there may be competition between precipitation and adsorption in the removal of have metals.

**Fig. 2:**
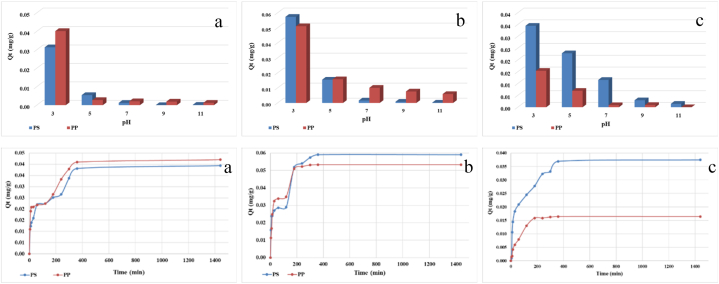
The effect of pH (a) Cd, (b) Ni and (c) Pb and contact time of (d) Cd^2+^, (e) Ni^2+^, and (f) Pb^2+^ on the retention capacity of heavy metals through adsorption on microplastics (PS and PP).

At lower pH values (acidic conditions), the microplastic surfaces tend to be positively charged due to the protonation of the functional groups promoting the adsorption of negatively charged metal ions through electrostatic attraction. As pH increases, microplastic surfaces become more negatively charged, potentially leading to reduce adsorption capacity for metal ions. Furthermore, at higher pH values, metal ions such as Cd, Ni, and Pb can precipitate as hydroxides, decreasing their availability for adsorption by microplastics. Therefore, lower pH values enhance adsorption due to increased surface charge, while higher pH values may limit adsorption due to ion precipitation and competition between precipitation and adsorption processes (Fig. 2 (a), (b), and (c)).

Experimental findings, revealed variation in the adsorption capacity of polystyrene and polypropylene microplastics for different heavy metal ions. Specifically, the adsorption capacity for cadmium was higher on polypropylene compared to polystyrene, possibly due to the surface properties and chemical composition of polypropylene, favoring Cd ion adsorption. Conversely, the adsorption capacities of nickel and lead on polystyrene were higher than on polypropylene, potentially related to surface characteristics of polystyrene creating a more favorable environment for the Ni and Pb ions.

The selection of pH 3 for the experiments alignes with previous research and literature findings [20], as acidic pH conditions have been shown to enhance the adsorption capacity of microplastics for metal ions. By choosing a pH value of 3, the study aims to maximize the adsorption potential of microplastics for Cd, Ni, and Pb ions.

### 3.3 Influence of the interaction time

In addition to pH, the contact time between metal ions and microplastics significantly impacts the adsorption process. Longer contact time allows for greater interactions between metal ions and microplastic surfaces, leading to retention through adsorption. In this study, a fixed contact time of 24 h was maintained to achieve equilibrium between the metal ions and microplastics, ensuring that the adsorption capacity reaches a plateau. Over the 24 h, metal ions (Cd^2+^, Ni^2+^, and Pb^2+^) in the solution interact with microplastics (PS and PP), attaching themselves to the surface. As time progresses, the adsorption process reaches saturation, stabilizing the adsorption capacity of microplastics.

The experimental results indicate that both PS and PP microplastics have the ability to adsorb Cd^2+^ ions from synthetic aqueous solutions, with comparable adsorption capacities of 0.038 mg/g for PS and 0.041 mg/g for PP (Fig. 2d). The observed increase in Cd^2+^ ions adsorbed per gram of microplastic over time indicates a two-stage adsorption process. The initial stage shows a significant increase in adsorption within the first 60 min, indicating numerous active sites available for Cd^2+^ ion binding. In the second stage, the adsorption rate slows as active sites become occupied, limiting further Cd^2+^ ion adsorption. The rapid adsorption process demonstrates the potential of microplastics to actively participate in removing Cd^2+^ ions from aqueous solutions. However, it is important to consider that the experimental conditions were conducted in a controlled laboratory setting. Real-world environmental conditions may differ, and other factors such as the presence of other ions or organic matter could influence the adsorption process.

The adsorption of Ni on both types of microplastics follows a similar pattern, with decreasing concentrations as contact time increases, reaching equilibrium after 24 h (Fig. 2e). Maximum adsorption capacities were measured at 0.059 mgNi/g for PS and 0.053 mgNi^2+^/g for PP. The two-stage adsorption process involves an initial rapid increase in Ni^2+^ ions retained per gram of microplastic, followed by a slower stage as active sites become occupied. PP microplastics exhibit faster equilibrium compared to PS, indicating a higher affinity and faster adsorption rate for Ni^2+^ ions. Both PS and PP microplastics demonstrate the ability to adsorb Ni^2+^ ions, with PS exhibiting higher adsorption capacities.

For Pb^2+^ ions, lower maximum retention capacities were observed on both microplastics, with maximum values of 0.037 mgPb^2+^/g for PS and 0.016 mgPb/g for PP (Fig. 2f). Adsorption trends of Pb^2+^ on microplastics also follow a two-stage pattern, with rapid occupation of active sites followed by a slower stage reaching equilibrium (Fig. 2c). PP microplastics reach equilibrium faster (after 180 min) than PS (after 360 min) suggesting a higher affinity and faster adsorption rate for Pb^2+^ ions. The lower adsorption capacities of both microplastics for Pb^2+^ ions compared to Cd^2+^ and Ni^2+^ ions. This could be attributed to the different chemical properties and interactions between Pb^2+^ ions and the surface functional groups of the microplastics. It is important to note that the adsorption process of Pb^2+^ ions on microplastics follows a similar two-stage pattern as observed for Cd^2+^ and Ni^2+^ ions indicate differences in affinity, likely due to varying chemical properties and interactions between Pb^2+^ ions and microplastic surface functional groups.

The discrepancy in Pb^2+^ adsorption amounts between (PS) and polypropylene (PP) microplastics can be attributed to their distinct surface properties and chemical compositions microplastics. The more rapid equilibrium attainment for PP indicates a higher affinity and faster adsorption rate for Pb^2+^ ions. These findings demonstrate the importance of considering unique microplastic characteristics in understanding heavy metal ion adsorption behavior.

Overall, both PS and PP microplastics show the ability to adsorb heavy metal ions, but specific adsorption capacities and behaviors vary depending on the metal ion and microplastic type.

### 3.4 Adsorption isotherms

The adsorption isotherm is a valuable tool for understanding the distribution of pollutants between the solid and liquid phases when adsorption equilibrium is reached. Two commonly used models for interpreting adsorption isotherms are the Langmuir model based on monolayer adsorption with no interaction forces among adsorbed molecules [44], and the Freundlich, an empirical equation that does not rely on specific assumptions. Several researchers have investigated the adsorption of toxic metals on microplastics and have reported that the Langmuir model effectively describes the adsorption of toxic metals in a monolayer manner on the microplastic surface [45,46]. However, other studies have argued that the Freundlich model provides a better fit for these adsorption processes [47]. According to the Freundlich model, adsorption occurs in a multilayer fashion on the heterogeneous microplastic surface, with pollutant molecules initially occupying high-energy adsorption sites and subsequently diffusing towards low-energy adsorption sites [48]. Importantly, there are also studies demonstrating that both the Langmuir and Freundlich models are effective in describing the adsorption isotherms [49,50].

**Fig. 3:**
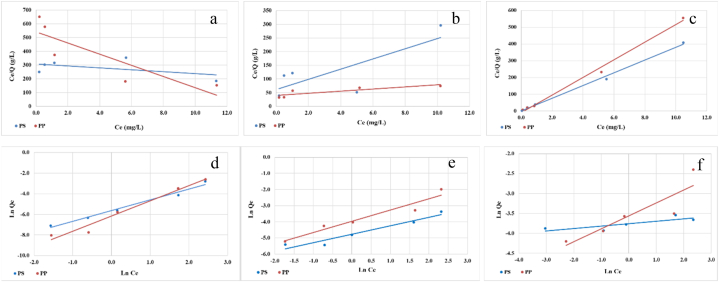
Langmuir adsorption isotherm corresponding to (a) Cd^2+^ (b) Ni^2+^ and (c) Pb^2+^; Frendlich adsorption isotherm corresponding to (d) Cd^2+^, (e) Ni^2+^, and (f) Pb^2+^.

In this study, adsorption isotherms were examined by varying concentrations in the range of 0.2–0.5-1-5-10 mg/L. Analysis using the Langmuir and Freundlich isotherm models revealed differences in regression coefficients. For Cd^2+^ and Ni^2+^, the Freundlich model showed higher regression coefficients, indicating a better fit compared to the Langmuir model (Fig. 3). On the other hand, the Langmuir model provided a better description of Pb^2+^ adsorption on both types of microplastics. These findings align with previous research indicating that the Freundlich model is suitable for depicting multilayer adsorption mechanism on heterogeneous surfaces [[51], [52], [53], [54], [55]], when describing heavy-metal sorption on microplastics.

The higher regression coefficients obtained for Cd^2+^ and Ni^2+^ with the Freundlich model indicate a stronger affinity for multiple layers on the microplastic surface, likely due to various available adsorption sites and stronger metal-microplastic interactions. In contrast, Pb^2+^ exhibited a better fit with the Langmuir isotherm, suggesting a monolayer adsorption mechanism where a limited number of equivalent adsorption sites are sequentially filled until saturation.

The observed multilayer adsorption behavior for Cd^2+^ and Ni^2+^ on the microplastics may be attributed to the presence of diverse adsorption on the heterogeneous microplastic surface, including both hydrophobic and hydrophilic sites. The Freundlich model, which considers the heterogeneity and multilayer adsorption, effectively describes the adsorption behavior of Cd^2+^ and Ni^2+^ on microplastics. These findings highlight the complex surface properties of microplastics and their role in providing diverse adsorption environments for heavy metals, supporting the applicability of the Freundlich isotherm in understanding multilayer adsorption processes.

The Freundlich isotherm model provided the best description of the Cd^2+^ adsorption process on both PS and PP microplastics in the conducted experiments. The Freundlich isotherm characterizes non-ideal and reversible adsorption, allowing for interactions between sorbed molecules and suggesting adsorption on heterogeneous surfaces with high-affinity sites being occupied first. The analysis of Cd2+ adsorption onto PS and PP microplastics using the Freundlich model yielded parameters including K_F_ = 280 mg Cd/g PS and K_F_ = 475 mg Cd/g PP; constants n = 0.964 for Cd adsorption on PS and n = 0.679 for Cd adsorption on PP; and correlation coefficients R^2^ = 0.9796 (PS) and R^2^ = 0.9696 (PP) (Fig. 2f). The obtained value of 1/n higher than 1 indicated that Cd^2+^ adsorption onto PS and PP is unfavorable over the studied concentration range. The unfavorable sorption behavior could be related to the competition for available adsorption sites on the microplastics between Cd^2+^ ions and other ions or molecules in the aqueous solution. In complex environmental systems, there may be various other substances present that can compete with Cd^2+^ ions for adsorption sites. These competing species could include other metal ions, organic compounds, or even dissolved organic matter. Secondly, the unfavorable sorption could be influenced by the inherent characteristics of Cd^2+^ ions. Cd^2+^ ions may possess a lower affinity for the microplastics compared to other metal ions, which could lead to less efficient adsorption. Additionally, Cd^2+^ ions may have a higher tendency to exist in solution as hydrated species, which could hinder their interaction with the microplastic surface. This weaker interaction between Cd^2+^ ions and microplastics could contribute to the observed unfavorable sorption behavior. Furthermore, the unfavorable sorption could be influenced by the surface properties of the microplastics themselves. Microplastics can have varying surface characteristics, such as hydrophobicity, surface charge, or presence of functional groups, which can affect the adsorption behavior of Cd^2+^ ions. If the microplastics used in the experiments have surfaces that are less favorable for Cd^2+^ adsorption, it can result in an unfavorable sorption process. It is important to note that the determination of unfavorable sorption does not necessarily imply that the adsorption process is ineffective or without significance. Unfavorable sorption simply indicates that the adsorption of Cd^2+^ ions onto PS and PP microplastics is not as efficient or as favorable as it could be.

For the Ni^2+^ adsorption experiments on PS and PP microplastics, the Freundlich isotherm exhibited higher correlation coefficients (R^2^) above 0.9, indicating the best fit for describing the adsorption processes (Table 1). Thus, the adsorption of Ni^2+^ on PS and PP surfaces occurs in a multilayer manner. The values of the constant 1/n between 0 and 1 (0 < 1/n < 1) in both cases, suggested favorable adsorption behavior, with strong bonding between the adsorbed Ni ions and microplastics. The multilayer adsorption behaviors implied by the Freundlich model indicate that Ni ions can form multiple layers on the microplastic surfaces due to the presence of active sites or functional groups, enhancing Ni ion adsorption capacity. For Ni adsorption on PS and PP microplastics, the Langmuir isotherm, with lower correlation coefficients, may not accurately represent the adsorption process, suggesting that monolayer adsorption is not the dominant mechanism. Instead, the Freundlich isotherm, which describes non-ideal adsorption on heterogeneous surfaces, provides a better fit to the experimental data. The multilayer adsorption behaviours could be due to the presence of various functional groups, surface irregularities, or varying properties on the microplastic surfaces, leading to a greater capacity for Ni ion adsorption.

**Table 1:** Parameters of Langmuir and Freundlich isotherms for Cd^2+^, Ni^2+^ and Pb^2+^ adsorption.

Metal	Adsorbant	Langmuir Isotherm Constants			Freundlich Isotherm Constants			
		Q_max_ (mg/g)	K_L_ (L/mg)	R^2^	K_F_ (mg/g)	n	1/n	R^2^
Cd^2+^	PS	0.142	0.023	0.2065	280	0.964	1.0376	0.9796
	PP	0.015	0.084	0.4496	475	0.679	1.4723	0.9696
Ni^2+^	PS	0.054	0.302	0.6033	89	1.191	0.5239	0.9364
	PP	0.259	0.066	0.7324	57	1.444	0.6943	0.9309
Pb^2+^	PS	0.026	83.4	0.9958	43	16.7	0.0597	0.6643
	PP	0.019	5.41	0.9935	50	3.09	0.3237	0.7883

In contrast, the Langmuir isotherm provided a better description of the Pb^2+^ adsorption process on both PS and PP microplastics, with higher correlation R^2^ values close to 1 coefficients (0.9935 and 0.9958 respectively), indicating a strong correlation (Table 1). The monolayer adsorption mechanism described by the Langmuir model suggests that Pb^2+^ ions occupy specific sites on the microplastic surface. On the other hand, the lower R^2^ values obtained for the Freundlich isotherm indicate a weaker correlation with the experimental data. The deviations between the experimental data and the Freundlich model could be attributed to factors such as heterogeneous surfaces, varying surface properties, or interactions between Pb^2+^ ions and the microplastic matrix that are not well-described by the model.

The RL factor, indicating the favorability of adsorption, fell between 0 and 1, suggesting a favorable adsorption process for both PS and PP microplastics. As Pb concentration decreased, the RL factor increased, indicating higher adsorption onto the microplastics likely due to increased surface area and binding sites at lower concentrations.

Previous studies in the literature have also shown that both Langmuir and Freundlich models effectively describe the adsorption of various metals on microplastics, supporting the findings of this study. The favorable adsorption behaviors observed for Ni^2+^ and Pb^2+^ ions on PS and PP microplastics suggest strong interactions between the metal ions and microplastic surfaces, highlighting the potential of microplastics as effective adsorbents for heavy metal removal from contaminated water sources [11,56,57].

### 3.5 Adsorption kinetics

**Fig. 4:**
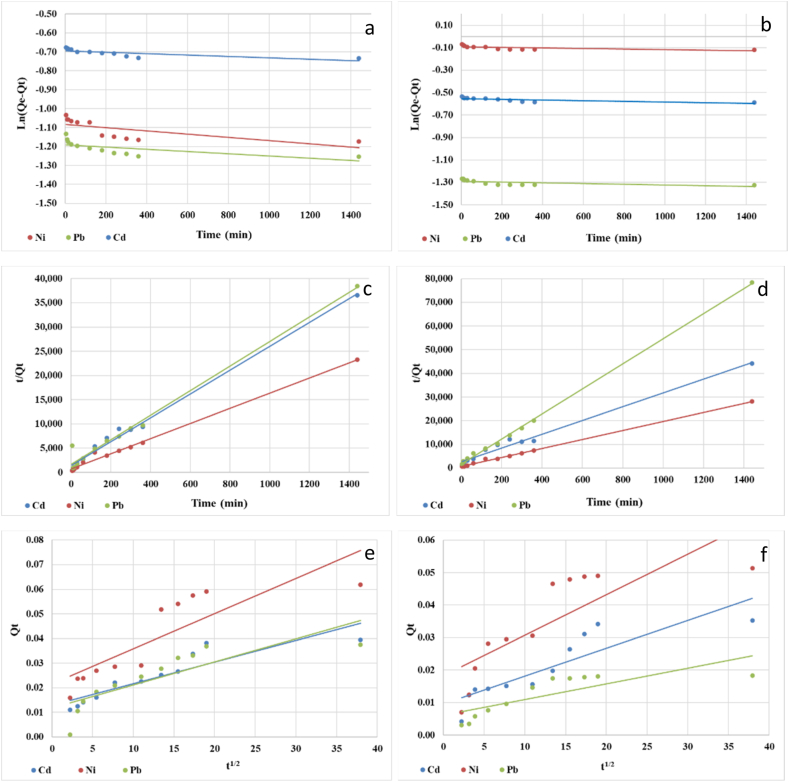
Pseudo-first-order kinetics of adsorption of heavy metals on (a) PS, and (b) PP; pseudo-second-order kinetics of adsorption of heavy metals on (c) PS, and (d) PP; intra-particle diffusion kinetics of adsorption of heavy metals on (e) PS, and (f) PP.

The results of this study provide valuable insights into the adsorption kinetics of heavy metals on PP and PS microplastics. By employing three kinetic models (Fig. 4), (1) the pseudo-first-order kinetic model, (2) the pseudo-second-order kinetic model, and (3) the intra-particle diffusion model, a comprehensive understanding of the competitive mechanism between the liquid and solid phases during the adsorption process was achieved [58]. The experimental data and the corresponding correlation coefficients (R^2^) for each model can be found in Table 2.

**Table 2:** Parameters of pseudo-first order, pseudo-second order and intra-particle kinetic models.

Metal	MPs	Qe, exp (mg/g)	Pseudo-first order			Pseudo-second order			Intra-particle difusion	
			Qe, calc (mg/g)	k_1_ (min^−1^)	R^2^	Qe, calc (mg/g)	k_2_ (g/mg x min)	R^2^	k_id_ (g/mg × min)	R^2^
Cd^2+^	PS	0.040	0.500	0.2778 × 10^−7^	0.5549	0.041	0.4370	0.9916	0.009	0.8102
	PP	0.031	0.575	0.2083 × 10-^6^	0.5233	0.034	0.3307	0.9864	0.0009	0.7620
Ni^2+^	PS	0.057	0.339	0.6250 × 10^−7^	0.4582	0.064	0.3367	0.9923	0.0014	0.7212
	PP	0.051	0.912	0.2083 × 10^−7^	0.3627	0.053	0.5434	0.9980	0.0014	0.6502
Pb^2+^	PS	0.034	0.304	0.4167 × 10^−7^	0.4101	0.039	0.3865	0.9836	0.0014	0.6882
	PP	0.015	0.275	0.2083 × 10^−7^	0.3312	0.019	0.6338	0.9988	0.0018	0.6311

The pseudo-second-order kinetic model was found to be the most suitable for describing the adsorption behaviour of heavy metals on microplastics. This suggests that the adsorption rate is primarily influenced by the adsorbate concentration on the microplastic surface, highlighting the importance of surface chemistry and available adsorption sites. The significantly higher correlation coefficients obtained with the pseudo-second-order model compared to the pseudo-first-order model suggest a better fit of the experimental data, indicating a second-order reaction mechanism where the rate of adsorption is dependent on the square of the adsorbate concentration. This insight can be useful in predicting and modelling the adsorption behaviour of heavy metals on microplastics in real-life scenarios. Variations in the calculated pseudo-second-order rate constants (K_2_) among different heavy metals and microplastic types indicate differences in adsorption capacities and reactivity.

**Fig. 5:**
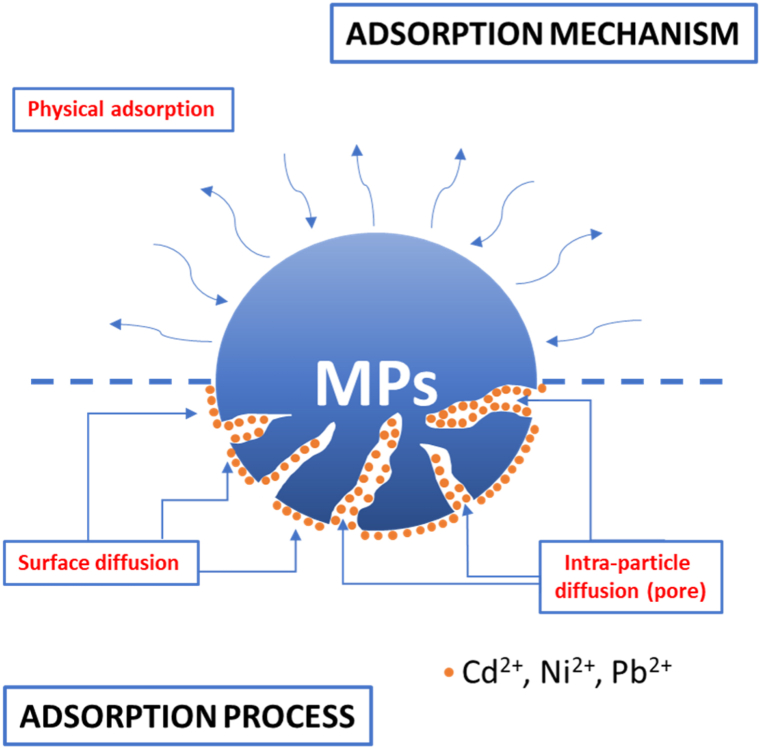
Schematic representation of possible metal adsorption mechanisms by MPs.

The analysis of the intra-particle diffusion graph in Fig. 4e and f provides valuable insights into the adsorption process of toxic metals on microplastic particles [59], demonstrating three distinct stages. The initial stage involves rapid occupation of active sites by the toxic metal ions attributed to covalent and van der Waals forces, facilitating efficient binding to the microplastic surface. As the adsorption progresses, metal ions diffuse into the microplastic pores, overcoming diffusion resistance within the particles. The final stage reaches equilibrium between adsorption and desorption, with decreased adsorption rates and metal ions diffused into the microplastic particles. The k_id_ constants (situated between 0.0014 and 0.009), representing the rate of intra-particle diffusion, provide quantitative information with higher constants indicating faster diffusion. the findings suggest that the pseudo-second-order kinetic model accurately describes the adsorption behaviours of heavy metals on PP and PS microplastics, offering insights into predicting adsorption capacities and designing effective removal processes for heavy metal contaminants using microplastics. The intra-particle diffusion analysis provides a detailed understanding of the adsorption process dynamics, shedding light on the mechanisms involved in heavy metal adsorption on microplastic particles. A schematic representation of the heavy-metal ions adsorption mechanism onto the MPs surface is shown in Fig. 5.

Understanding the various stages of the adsorption process and the significance of intra-particle diffusion is essential for devising efficient strategies to address the environmental consequences of toxic metals associated with microplastic pollution. This comprehension can aid in optimizing adsorption procedures, enhancing removal efficiency, and formulating suitable measures to mitigate the adverse impacts of these contaminants on ecosystems.

### 3.6 Coexistence adsorption

**Fig. 6:**
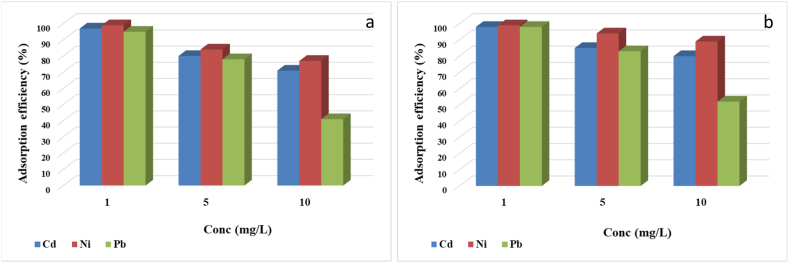
Adsorption efficiency of Cd^2+^, Ni^2+^, and Pb^2+^ onto: (a) PS and (b) PP.

The comparison of adsorption capacities for Cd, Ni, and Pb ions on polystyrene and polypropylene microplastics demonstrates distinct behaviours. Fig. 6 illustrates the adsorption efficiency of the three heavy metals on the microplastics. The adsorption efficiency reaches its peak for Cd, Ni, and Pb when the mixed adsorbent concentration is at 1 mg/L, with a decline in efficiency as the adsorbate concentration increases. At lower adsorbate concentrations, the adsorption effects on the three heavy metals can be considered close to saturation, while at higher concentrations, the removal efficiency diminishes.

Moreover, the coexistence of various metals can initially enhance the adsorption of Pb at low concentrations, but as the concentration of adsorbate rises, the coexisting system may inhibit the adsorption onto the microplastics. This phenomenon is consistent with the findings of Taha et al. who observed in their study on multicomponent adsorption of Pb, Cd, and Ni by microwave-functionalized cellulose that the presence of Cd and Ni can impede the absorption of Pb, with Pb exhibiting the most significant effect on adsorption capacity [60]. Such observations suggest that the adsorption mechanism of different heavy metals and their interactions in a multi-component system can influence their adsorption onto microplastics, yielding varied outcomes based on distinct adsorption mechanisms and reaction conditions.

## 4 Conclusion

This study aimed to analyze and discuss the adsorption mechanism of Cd^2+^, Ni^2+^, and Pb^2+^ by polystyrene (PS) and polypropylene (PP) microplastics to better understand their potential environmental implications. Our findings revealed several important insights into the adsorption behaviour of heavy metals on microplastics. The results demonstrated that the adsorption behaviour reached equilibrium within a relatively short duration of contact time, with all metals showing equilibrium adsorption within 6 h. This indicates that microplastics have a high affinity for heavy metals and can efficiently adsorb them from the surrounding environment. The FTIR analysis and surface morphology observations indicate that physical interactions, along with surface properties, significantly contribute to the adsorption of heavy metals onto microplastics. Moreover, it was observed that the adsorption capacity of heavy metals was significantly influenced by solution pH, contact duration, and initial metal concentration. These factors can affect the adsorption behaviour, indicating the need to consider environmental conditions when assessing the fate and transport of microplastics and heavy metals in aquatic ecosystems.

The Langmuir adsorption isotherm model provided a better fit to the experimental data compared to the Freundlich model, suggesting monolayer adsorption and homogeneous surface adsorption of heavy metals on microplastic surfaces. This information can be useful in modelling and predicting the adsorption behaviour of heavy metals on microplastics in real-world scenarios. Furthermore, the pseudo-second-order kinetic model was found to describe the adsorption process more accurately than the pseudo-first-order kinetic model, indicating that the adsorption rate is primarily influenced by the adsorbate concentration on the microplastic surface.

Overall, this study contributes to our understanding of the adsorption mechanism of heavy metals on microplastics and provides insights into their behaviour in aquatic environments. The findings emphasize the need for effective strategies to mitigate microplastic and heavy metal pollution, protecting the health of aquatic ecosystems. Future research should explore the long-term effects of microplastics and heavy metals, including their potential impacts on organisms, food chains, and overall ecosystem health. Additionally, further studies on the surface modifications of microplastics and their influence on adsorption behaviour will be crucial to enhance our understanding and develop effective mitigation strategies.

## Ethics declarations

Review and approval by an ethics committee was not required for this study because this article does not involve any direct experimentation and studies on living beings.

## Data availability statement

The data generated and analyzed during this work will be presented on request.

## CRediT authorship contribution statement

**Anda-Gabriela Tenea:** Writing – review & editing, Writing – original draft, Validation, Formal analysis, Data curation. **Cristina Dinu:** Writing – review & editing, Writing – original draft, Visualization, Supervision, Methodology, Data curation. **Paul Alexandru Rus:** Writing – review & editing, Writing – original draft, Resources, Funding acquisition, Formal analysis. **Ioana Alexandra Ionescu:** Writing – review & editing, Writing – original draft, Visualization, Software, Investigation, Formal analysis. **Stefania Gheorghe:** Writing – review & editing, Writing – original draft, Supervision, Resources, Project administration, Conceptualization. **Vasile Ion Iancu:** Writing – review & editing, Writing – original draft, Visualization, Software, Investigation, Formal analysis, Conceptualization. **Gabriela Geanina Vasile:** Writing – review & editing, Writing – original draft, Visualization, Validation, Supervision, Methodology, Conceptualization. **Luoana Florentina Pascu:** Writing – review & editing, Writing – original draft, Visualization, Supervision, Investigation, Conceptualization. **Florentina Laura Chiriac:** Writing – review & editing, Writing – original draft, Visualization, Validation, Supervision, Resources, Project administration, Methodology, Data curation, Conceptualization.

## Declaration of competing interest

The authors declare that they have no known competing financial interests or personal relationships that could have appeared to influence the work reported in this paper.
